# Single-Lung Ventilation in Infants for Surgical Repair of Coarctation of The Aorta Without Cardiopulmonary Bypass

**DOI:** 10.21470/1678-9741-2022-0424

**Published:** 2024-04-15

**Authors:** Ling-Shan Yu, Si-Jia Zhou, Xiu-Hua Chen, Jing Wang, Zeng-Chun Wang

**Affiliations:** 1 Department of Cardiac Surgery, Fujian Children’s Hospital (Fujian Branch of Shanghai Children’s Medical Center), College of Clinical Medicine for Obstetrics & Gynecology and Pediatrics, Fujian Medical University, Fuzhou, China

**Keywords:** Cardiopulmonary Bypass, Aortic Coarctation, One-Lung Ventilation, Postoperative Complications, Hypoxia

## Abstract

**Objective:**

To investigate the effect of improving the operative field and postoperative atelectasis of single-lung ventilation (SLV) in the surgical repair of coarctation of the aorta (CoA) in infants without the use of cardiopulmonary bypass (CPB).

**Methods:**

This was a retrospective cohort study. The clinical data of 28 infants (aged 1 to 4 months, weighing between 4.2 and 6 kg) who underwent surgical repair of CoA without CPB from January 2019 to May 2022 were analyzed. Fourteen infants received SLV with a bronchial blocker (Group S), and the other 14 infants received routine endotracheal intubation and bilateral lung ventilation (Group R).

**Results:**

In comparison to Group R, Group S exhibited improved exposure of the operative field, a lower postoperative atelectasis score (P<0.001), reduced prevalence of hypoxemia (P=0.01), and shorter durations of operation, mechanical ventilation, and ICU stay (P=0.01, P<0.001, P=0.03). There was no difference in preoperative information or perioperative respiratory and circulatory indicators before SLV, 10 minutes after SLV, and 10 minutes after the end of SLV between the two groups (P>0.05). Intraoperative bleeding, intraoperative positive end-expiratory pressure (PEEP), and systolic pressure gradient across the coarctation after operation were also not different between the two groups (P>0.05).

**Conclusion:**

This study demonstrates that employing SLV with a bronchial blocker is consistent with enhanced operative field, reduced operation duration, lower prevalence of intraoperative hypoxemia, and fewer postoperative complications during the surgical repair of CoA in infants without the use of CPB.

## INTRODUCTION

**Table:** 

Abbreviations, Acronyms & Symbols	
CI	= Confidence interval
CoA	= Coarctation of the aorta
CPB	= Cardiopulmonary bypass
CT	= Computed tomography
FiO_2_	= Fraction of inspired oxygen
ICU	= Intensive care unit
HR	= Heart rate
MAP	= Mean arterial blood pressure
OR	= Odds ratio
PaO_2_	= Partial pressure of oxygen
PEEP	= Positive end-expiratory pressure
PETCO_2_	= End-tidal carbon dioxide partial pressure
PPEAK	= Peak pressure
SLV	= Single-lung ventilation
SPSS	= Statistical Package for the Social Sciences
VT	= Volume

Coarctation of the aorta (CoA) is the narrowing of the aortic segment^[1,2]^. Surgical correction is still the preferred treatment for most patients, especially infants and young children^[3]^. In the conventional surgical repair of CoA without cardiopulmonary bypass (CPB), routine endotracheal intubation and bilateral lung ventilation are usually used, which affects the surgical field of vision, easily causes lung injury, and increases the incidence of postoperative atelectasis^[4,5]^. In recent years, bronchial blockade for single-lung ventilation (SLV) technology has been applied gradually, which can improve the operation field exposure and has no apparent influence on hemodynamics^[6,7]^. At present, there is no large sample studies about the application of bronchial blockade SLV in the surgical repair of CoA in infants without CPB.

Lung ultrasound is a noninvasive, portable, accurate, and reliable method for bedside diagnosis of postoperative atelectasis in children^[8]^. This retrospective study analyzed the application of bronchial blockade SLV in the surgical repair of CoA in infants without CPB, combined with lung ultrasound to analyze its effects and provides a basis for its clinical application.

## METHODS

This study aimed to analyze the effects of using bronchial blockade SLV in the surgical repair of CoA in infants without CPB combined with lung ultrasound.

The Ethics Committee of our hospital approved this study. Written informed consent was obtained from the parents/guardians of the patients.

The sample size was determined with PASS 11 software (NCSS, LLC, Kaysville, UT). Atelectasis and lung collapse scores were our primary compared objectives, and these parameters were used to calculate the sample size. In the preliminary survey, data from 7 patients were collected in Group S and Group R, respectively. The proportion of 1 score in the atelectasis score for Group S was 5 (71.4%), while for Group R it was 1 (14.3%). The ratio between the two groups was 1:1, with an alpha value of 0.05, and the power was set to 0.90. The calculated sample size was 14 in each group. The proportion of 1 score in the lung collapse score of Group S was 6 (85.7%). and in Group R it was 1 (14.3%). In the same way, we used the lung collapse score to calculate the sample size. The calculated sample size was 9 in each group. As a result, the total sample size required was 14^[9]^.

The inclusion criteria were defined as follows: (1) confirmation through echocardiography and computed tomography angiography that patients had simple CoA, consistent with the indications for surgical repair of CoA without CPB (characterized by delayed or absent femoral pulses, an arm/leg systolic blood pressure difference of 20 mmHg, and significant hypertension or congestive heart failure)^[10]^; (2) no obvious anesthesia or surgical contraindications (such as severe pulmonary hypertension or infection); (3) American Association of Anesthesiologists (ASA) class II-III; and (4) age under one year (patients included in this study used a bronchial blocker outside the endotracheal tube, which applies for patients under one year old in our center). Exclusion criteria included the following: (1) complex CoA or other cardiac abnormalities requiring concurrent surgical correction with CPB, except for patent ductus arteriosus (PDA); (2) tracheal tube size <3.0 mm (due to the inability of fiberoptic bronchoscopy [FB] to pass through the endotracheal tube); (3) intubation difficulties; (4) severe pulmonary infection and respiratory insufficiency (as these conditions predisposed patients to hypoxemia during SLV); and (5) alterations in ventilation patterns during operation. All patients completed the routine preoperative examinations, and relevant data are shown in Table 1.

**Table 1:** General preoperative conditions of patients.

	Group S (n=14)	Group R (n=14)	T	*P*
Age (months)	2.7±0.8	2.6±1.0	0.21	0.84
Gender (male/female)	6-ago.	6-ago.	/	/
Body weight (kg)	5.0±0.6	5.2±0.6	-0.93	0.36
Systolic pressure gradient across the coarctation before operation (mmHg)	45.6±9.4	47.0±9.7	-0.38	0.71

This was a retrospective cohort study. Sixty-two infants underwent CoA repair from January 2019 to May 2022. Clinical data of these patients were collected from the electronic medical record system. According to the inclusion and exclusion criteria and employing a simple match for age and gender, 14 infants received SLV with a bronchial blocker in Group S. Another 14 infants who underwent routine endotracheal intubation and bilateral lung ventilation were included in Group R. The choice of anesthesia protocol was determined by the anesthesiologist, surgeon, and the preferences of the patient’s family.

The purpose of our study was to analyze the effects of improving the operative field and reducing postoperative atelectasis of SLV in the surgical repair of CoA in infants without CPB.

In the operating room, all patients underwent monitoring of peripheral blood oxygen saturation, noninvasive blood pressure, and electrocardiography. Anesthesia induction consisted of an intravenous administration of midazolam 0.1 mg/kg for sedation, sufentanil 1 µg/kg for analgesia, and rocuronium 0.6 mg/kg for muscle relaxation. After muscle relaxation, patients in Group S underwent the following steps: first, placement of a bronchial blocker (5F with an external diameter of 1.7 mm; Hangzhou Tanpa Medical Technology, Hangzhou, Zhejiang, China) into the glottis using a video laryngoscope; second, endotracheal intubation (the bronchial blocker was placed outside the endotracheal tube); and third, confirmation of the positions of the endotracheal tube and the bronchial blocker by inserting a FB into the endotracheal tube. The position of the endotracheal tube was approximately 1-2 cm proximal to the carina, and the cuff of the bronchial blocker was guided into the main bronchus at the surgical site. Arterial blood pressure was monitored by the right radial artery and femoral artery, and central venous pressure was monitored through subclavian vein puncture. Anesthesia maintenance consisted of continuous intravenous infusion of sufentanil 1-2 µg/kg/h for analgesia, midazolam 0.05 mg/kg/h for sedation, and rocuronium 0.6 mg/kg/h for muscle relaxation.

The patients were placed in the right decubitus position, and a left posterolateral thoracotomy at the third or fourth intercostal space was performed. In Group S, the location of the bronchial blocker was reconfirmed by FB before the skin incision. The cuff of the bronchial blocker was filled with 1-2 mL of air to perform SLV when a skin incision was made. When the chest was closed, the patients restored bilateral lung ventilation. In Group R, preparation before anesthetic induction and the anesthetic procedure were the same as those in Group S. However, following muscle relaxation, routine endotracheal intubation and bilateral lung ventilation were performed.

The pressure control mode of mechanical ventilation was adopted in all patients. Respiratory parameters were set as follows: fraction of inspired oxygen (FiO₂) set between 50-100%, positive end-expiratory pressure (PEEP) at 3-5 cmH₂O, inspiratory/expiratory ratio (I:E) at 1:1.5, tidal volume (VT) set at 6-8 mL/kg, respiratory frequency (R) set between 25-35 times/min, and oxygen flow at 2-3 L/min. FiO₂ and respiratory frequency were adjusted according to blood gas analysis values. The end-tidal carbon dioxide partial pressure (PETCO₂) was intraoperatively maintained at 35-45 mmHg.

During the operation, the same group of surgeons unaware of patient grouping evaluated the exposure of the surgical field according to Javier H. Campos' lung collapse score^[11]^. After operation, the patients were moved from lateral to the supine position. The bronchial blocker was removed in Group S, and lung recruitment was performed in both groups (the peak inspiratory pressure was 30 cmH₂O for 15-20 s)^[12]^. Then, the patients were transferred to the intensive care unit (ICU) for further monitoring and treatment.

After the patients returned to the ICU, a routine lung ultrasound was performed according to the localization method described by Acosta et al.^[13]^ The two lungs were divided into anterior, lateral, and posterior areas based on the axillary front and posterior axillary line. Additionally, a horizontal division into upper and lower regions was made at the level of 1 cm above the nipple, with a total of 12 lung regions. Juxtapleural consolidation of different sizes and the B line were the two most common lung ultrasound signs^[14]^. These lung ultrasound signs were recorded. All lung ultrasound examinations were performed by an ICU physician proficient in pediatric lung ultrasound. Importantly, this physician was unaware of the patient grouping and was a part of our research team.

To minimize operator differences, all anesthetic procedures were performed by three cardiothoracic anesthesiologists at our center. Each anesthesiologist performed both types of procedures (Group S and Group R). The unified team of ICU physicians made decisions regarding tracheal tube removal and ICU discharge according to the patient's actual situation. Postoperative analgesia and sedation management were the same in both groups.

Data were collected and statistical analysis conducted, including the following parameters:

1) Patient characteristics (age, body weight, systolic pressure gradient across the coarctation before the operation).

2) Primary outcomes: prevalence of intraoperative hypoxemia (peripheral blood oxygen saturation <90%), degree of lung collapse^[11]^ (1 score, operative-side lung collapse with satisfactory exposure of the operative field without intervention, which did not affect the operation; 2 score, partial collapse of the operative-side lung, acceptable exposure after intervention without affecting the operation; 3 score, severe collapse of the operative-side lung; the exposure of the surgical field and operation were still seriously affected after intervention), and an atelectasis ultrasound score^[15]^ (degree of juxtapleural consolidation: 0 represents no consolidation; 1 represents minimal juxtapleural consolidation; 2 represents small-sized consolidation; 3 represents large-sized consolidation. B-lines: 0 represents fewer than three isolated B-lines; 1 represents multiple well-defined B-lines; 2 represents multiple coalescent B-lines; 3 represents white lung).

3) Secondary outcomes: mean arterial blood pressure (MAP), heart rate (HR), airway peak pressure (Ppeak), and the oxygenation index (PaO₂/FiO₂ ratio) were measured at these time points -- before SLV (T1), 10 minutes after SLV (T2), and 10 minutes after the end of SLV (T3); operation duration, mechanical ventilation duration and length of ICU stay; intraoperative bleeding (a small amount of bleeding was estimated using gauze weight. The weight of the wet gauze minus the weight of the dry gauze was used to evaluate the blood loss by the algorithm of 1 g to 1 mL), intraoperative PEEP, and the systolic pressure gradient across the coarctation after the operation (echocardiography was performed on the first postoperative day).

### Statistical Analysis

All data were entered into Microsoft Excel forms and analyzed using IBM SPSS statistical software, version 20.0. Independent continuous variables were analyzed by t-tests when data exhibited a normal distribution after testing (Shapiro-Wilk test) and expressed as mean±standard deviation (x±S). This included parameters such as age, body weight, systolic pressure gradient across the coarctation, MAP, HR, Ppeak, oxygenation index, intubation duration, operation duration, mechanical ventilation duration, length of ICU stay, intraoperative bleeding, and intraoperative PEEP. The chi-square test or Fisher’s exact probability method was used for categorical data. Counts and percentages describe the enumeration data. The Mann-Whitney U test was applied for non-normally distributed data. A *P*<0.05 was defined as statistically significant.

## RESULTS

### Patient Characteristics

There was no difference in preoperative clinical information (age/months, body weight/kg, systolic pressure gradient across the coarctation before the operation/mmHg), as evidenced by t-tests (T=0.21, *P*=0.84; T=-0.93, *P*=0.36; T=-0.38, *P*=0.71). This confirmed that the two groups of infants were comparable and homogeneous (Table 1).

### Primary Outcomes

The prevalence of hypoxemia was 1/14 (7.1%, OR=0.3, 95% CI: 1.1%-16.6%) in Group S and 4/14 (28.6%, OR=0.8, 95% CI: 1.5%-55.6%) in Group R. A significant difference was observed between the two groups (chi-square test or Fisher’s exact probability, χ^2^=5.89, *P*=0.01, OR=0.2, 95% CI: 2.7%-33.0%) (Table 2). The postoperative atelectasis score was lower in Group S than in Group R (Mann-Whitney U test, juxtapleural consolidation: mean rank=10.36 and 18.64, Z=-2.89, *P*<0.001; B-lines: mean rank=10.89 and 18.11, Z=-2.54, *P*<0.001) (Table 3). These results showed that SLV with a bronchial blocker might reduce the incidence of lung injury and increased the oxygen reserve. The exposure of the operative field was better in Group S than in Group R (Mann-Whitney U test, mean rank=9.14 and 19.86, Z=-3.77, *P*<0.001) (Table 3), which suggested that the degree of lung collapse was better in Group S.

**Table 2:** Perioperative data between the two groups.

	Group S (n=14)	Group R (n=14)	T/χ^2^	*P*
Intraoperative bleeding (mL)	17.3±2.1	20.7±5.3	-1.93	0.07
Systolic pressure gradient across the coarctation after operation (mmHg)	15.7±4.0	18.4±4.4	-2.05	0.06
Intraoperative PEEP (cmH₂O)	4.2±0.6	4.1±0.9	0.24	0.81
Prevalence of hypoxemia, n (%)	1 (7.1%)	4 (28.6%)	5.89	0.01
Operation duration (h)	1.4±0.5	2.0±0.3	-2.83	0.01
Mechanical ventilation duration (h)	3.8±0.8	5.7±1.0	-5.94	P<0.001
Length of ICU stay (h)	19.4±3.2	24.9±8.4	-2.29	0.03

ICU=intensive care unit; PEEP=positive end-expiratory pressure

**Table 3:** Degree of lung collapse and atelectasis score.

	Group S (n=14)	Group R (n=14)	Z	*P*	
Degree of lung collapse					
1 score, n (%)	12 (85.7%)	2 (14.3%)	-3.77	*P*<0.001	*P*<0.001
2 score, n (%)	2 (14.3%)	7 (50.0%)		0.10	
3 score, n (%)	0 (0.0%)	5 (35.7%)		0.04	
Juxtapleural consolidation					
0 score, n (%)	0 (0.0%)	0 (0.0%)	-2.89	/	*P*<0.001
1 score, n (%)	10 (71.4%)	2 (14.3%)		0.01	
2 score, n (%)	3 (21.4%)	8 (57.1%)		0.12	
3 score, n (%)	1 (7.1%)	4 (28.6%)		0.33	
B-lines					
0 score, n (%)	0 (0.0%)	0 (0.0%)	-2.54	/	*P*<0.001
1 score, n (%)	9 (64.3%)	2 (14.3%)		0.02	
2 score, n (%)	4 (28.6%)	9 (64.3%)		0.13	
3 score, n (%)	1 (7.1%)	3 (21.4%)		0.59	

### Secondary Outcomes

**Fig. 1:**
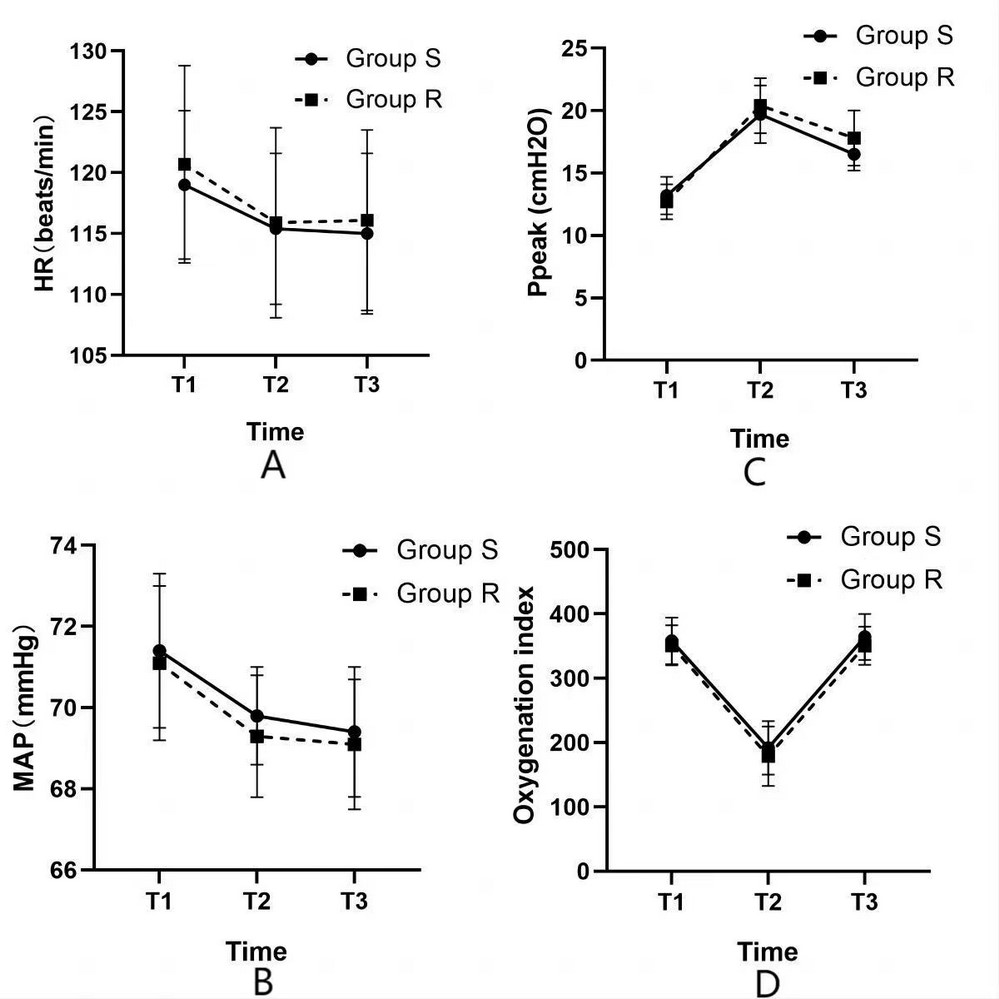
Intraoperative hemodynamic data (HR, MAP), Ppeak, and oxygenation index between the two groups. A, B, C, D show the HR, MAP, Ppeak, and oxygenation index between the two groups at T1, T2, and T3, respectively.

Perioperative hemodynamics (MAP/mmHg, HR/beats/min) and Ppeak (cmH₂O), as well as oxygenation index at T1, T2, and T3 between the two groups had no difference (t-tests, T1: MAP, T=0.49, *P*=0.82, HR, T=-0.64, *P*=0.72, Ppeak, T=0.91, *P*=0.37, oxygenation index, T=0.53, *P*=0.60; T2: MAP, T=0.96, *P*=0.85, HR, T=-0.19, *P*=0.79, Ppeak, T=-0.74, *P*=0.46, oxygenation index, T=0.78, *P*=0.44; T3: MAP, T=0.36, *P*=0.84, HR, T=-0.43, *P*=0.78, Ppeak, T=-1.85, *P*=0.08, oxygenation index, T=1.11, *P*=0.28) (Figure 1).

Intraoperative bleeding (mL), intraoperative PEEP (cmH₂O), and systolic pressure gradient (mmHg) across the coarctation after the operation also showed no difference between the two groups (t-tests, T=-1.93, *P*=0.07, T=0.24, *P*=0.81, T=-2.05, *P*=0.06) (Table 2).

Compared with Group R, Group S demonstrated shorter operation duration and mechanical ventilation duration (hours) (t-tests, T=-2.83, *P*=0.01; T=-5.94, *P*<0.001) and a reduced length of ICU stay (hours) (t-tests, T=-2.29, *P*=0.03) (Table 2).

## DISCUSSION

The ultrasound atelectasis score in Group S was lower than that in Group R, and the intraoperative prevalence of hypoxemia was 1/14 (7.1%) in Group S and 4/14 (28.6%) in Group R. These results show that SLV with a bronchial blocker in CoA surgery might reduce the incidence of lung injury induced by atelectasis and increased the oxygen reserve. During CoA surgery without CPB, patients were in the lateral decubitus position. The operative side lung tissue was manipulated and retracted during surgery when routine endotracheal intubation and bilateral lung ventilation were performed. For infants, small chest cavity, deep surgical position, narrow operative field, and dilated lungs might affect the surgical field of vision. To the exposure of the surgical field, surgeons would compress the lung tissue, which could lead to severe uneven ventilation in the lung and an increased susceptibility to atelectasis. While patients in Group S were under SLV with bronchial blockers, the operative side lung tissue collapsed, avoiding the compression and the lung injury induced by atelectasis.

The occurrence of atelectasis is significantly correlated with postoperative pulmonary complications^[16]^. In our study, patients in Group S had a lower incidence of postoperative atelectasis, resulting in shorter durations of mechanical ventilation and length of ICU stay compared to Group R. Postoperative atelectasis often required various physical therapies, such as prone positioning treatment, high-frequency oscillation ventilation, and higher PEEP to improve oxygenation status and open up alveoli, which could lead to prolonged hospital stays and increased hospitalization costs.

In recent years, the application of SLV with bronchial blockers has increased gradually in pediatric cases^[17]^. Hamid et al.^[18]^ reported the application of bronchial blockade SLV in CoA surgery in children at 19 months, demonstrating that it could improve the visibility of the surgical field. Fox et al.^[19]^ reported in an article on the perioperative management of CoA surgical patients that pulmonary isolation using double-lumen or bronchial blockers could significantly improve the visibility of the surgical field and assist exposure. These results were all consistent with our findings. In our study, the degree of lung collapse was better in Group S, which is more conducive to the exposure of surgical field, and the operation duration was also shorter in Group S than in Group R.

Lung ultrasound has the advantages of being noninvasive, portable, having no radiation and therefore no exposure to radiation, which can be used to evaluate atelectasis caused by various reasons^[20]^. A retrospective study found that the sensitivity of lung ultrasound in detecting atelectasis was greater than that of chest radiographs, with similar specificity^[21]^. Bouhemad et al.^[22]^ studied the changes in atelectasis in ventilator-associated pneumonia and found a significant positive correlation between computed tomography (CT) and lung ultrasound score. In this study, lung ultrasound was used to evaluate postoperative atelectasis after the patients returned to the ICU.

There was no difference in perioperative hemodynamics, Ppeak, oxygenation index, intraoperative bleeding, intraoperative PEEP, or systolic pressure gradient across the coarctation after the operation between the two groups. These results might suggest that SLV with a bronchial blocker was safe in the surgical repair of CoA without CPB in infants. Our results are also consistent with a study by Zhang, which illustrated the use of bronchial blocker SLV in minimally invasive cardiac surgery in adults^[23]^.

Placement of the bronchial blocker is as easy to perform as conventional tracheal intubation. All endotracheal intubation placements and bronchial blockers for infants at our institution were completed by three cardiothoracic anesthesiologists with extensive experience. All the patients in this study had no related complications, such as laryngeal edema or airway bleeding.

There were some limitations to this study. First, this was a retrospective study, not a prospective randomized controlled study, so there is the possibility of unseen biases. We have tried to reduce the generation of bias by simple matching the patients of two groups and the whole study process was performed by the unified team of physicians. Second, the sample size of this study was relatively small, and it was a single-center study, so the conclusion might be one-sided to some extent. The generalization of results might be an issue. However, there is no large sample article about the application of bronchial blockade SLV in the surgical repair of CoA in infants without CPB presently. This paper is still significant for guiding certain clinical situations. Future prospective, randomized controlled trials with large samples and multiple centers are needed to confirm the conclusions.

## CONCLUSION

In the present study, the application of SLV with a bronchial blocker consistently demonstrated enhancements in the operative field, a reduction in operation duration, and a decrease in the intraoperative prevalence of hypoxemia and postoperative complications in the surgical repair of CoA in infants without CPB.
